# Supplementary material for: Shear-Mediated Platelet Microparticles Demonstrate Phenotypic Heterogeneity as to Morphology, Receptor Distribution, and Hemostatic Function
Source: Int J Mol Sci. 2023 Apr 17;24(8):7386. doi: 10.3390/ijms24087386 (PMC10138836; doi:10.3390/ijms24087386)
Supplement: Supplementary file 1 [file ijms-24-07386-s001.zip › ijms-2297702-supplementary.pdf]

SUPPLEMENTARY MATERIALS

Full Title:
Shear-Mediated Platelet Microparticles Demonstrate Phenotypic Heterogeneity as to Morphology, Receptor Distribution, and Hemostatic Function

Yana Roka-Moiia[1], Kaitlyn Ammann[1], Samuel Miller-Gutierrez[1], Jawaad Sheriff[2], Danny Bluestein[2], Joseph E. Italiano[3], Robert C. Flaumenhaft[3,4], Marvin J. Slepian[1,2]

[1]Department of Medicine and Biomedical Engineering, Sarver Heart Center, University of Arizona, Tucson, AZ, USA; [2]Department of Biomedical Engineering, Stony Brook University, Stony Brook, NY, USA, [3]Boston Children's Hospital, Harvard Medical School, Boston MA, USA, [4]Beth Israel Deaconess Medical Center, Boston, MA, USA.

Running Head: **Phenotyping Shear-Mediated Platelet Microparticles**

Corresponding Author:

Marvin J. Slepian, MD
Sarver Heart Center, University of Arizona,
1501 N Campbell Ave, Tucson, AZ 85724
Email: slepian@email.arizona.edu
Fax: (520) 626-7625; Tell: (520) 626-4314

Major Resources Table

Antibodies

Target antigen	Vendor or Source	Catalog #	Final concentration	Lot #	Persistent ID / URL
CD41-APC, clone MEM-06	Thermo Fisher Scientific	MA1-19779	1:10	WI3374337	https://www.thermofisher.com/antibody/product/CD41-Antibody-clone-MEM-06-Monoclonal/MA1-19779
CD42a-FITC, clone GR-P	Thermo Fisher Scientific	11-0428-42	1:250	2279143	https://www.thermofisher.com/antibody/product/CD42a-Antibody-clone-GR-P-Monoclonal/11-0428-42
PAR1-AF488, clone 731115	R&D Systems	FAB3855G	1:20	ADTF0216101	https://www.rndsystems.com/products/human-par1-alexa-fluor-488-conjugated-antibody-731115_fab3855g
CD62P-APC, clone Psel.KO 2.3	eBioscience	17-0626-82	1:80	2460169	https://www.thermofisher.com/antibody/product/CD62P-P-Selectin-Antibody-clone-Psel-KO2-3-Monoclonal/17-0626-82
CD31-FITC clone WM-59	Thermo Scientific	17-0319-42	1:250	2119197	https://www.thermofisher.com/antibody/product/CD31-PECAM-1-Antibody-clone-WM-59-WM59-Monoclonal/17-0319-42
PSGL1-PE clone KPL-1	BD Pharmingen	556055	1:5	0044413	https://www.bdbiosciences.com/en-us/products/reagents/flow-cytometry-reagents/research-reagents/single-color-antibodies-ruo/pe-mouse-anti-human-cd162.556055

annexin V-FITC	eBioscience	BMS500FI-300	1:20	334226-000	https://www.fishersci.com/shop/products/anxnv-fit-apop-detn-kt-300t/509301
GP6-PE clone HY101	BD Pharmingen	565241	1:250	1046600	https://www.fishersci.com/shop/products/hu-platelet-gpvi-pe-hy101-50t/BDB565241
P2RY12-FITC clone S16001E	BioLegend	392108	1:20	B330902	https://www.biolegend.com/en-us/products/fitc-anti-human-p2ry12-antibody-19685#productCitations

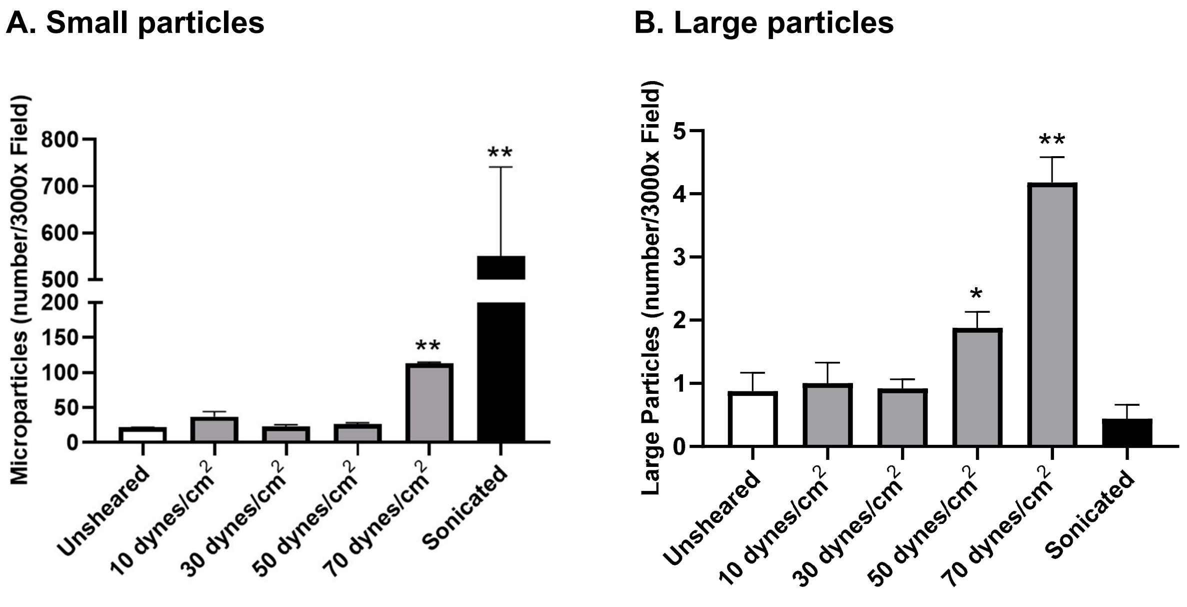

<u>Figure S1</u>. **High shear stress promotes generation of an increasing number of small (150 - 500 nm) and, to less extent, large (500 - 1000 nm) platelet-derived particles. Sonication promotes generation of small, but not large, particles: A** – small platelet-derived particles, **B** – large platelet-derived particles. ANOVA: * - $p < 0.05$; ** - $p < 0.01$.

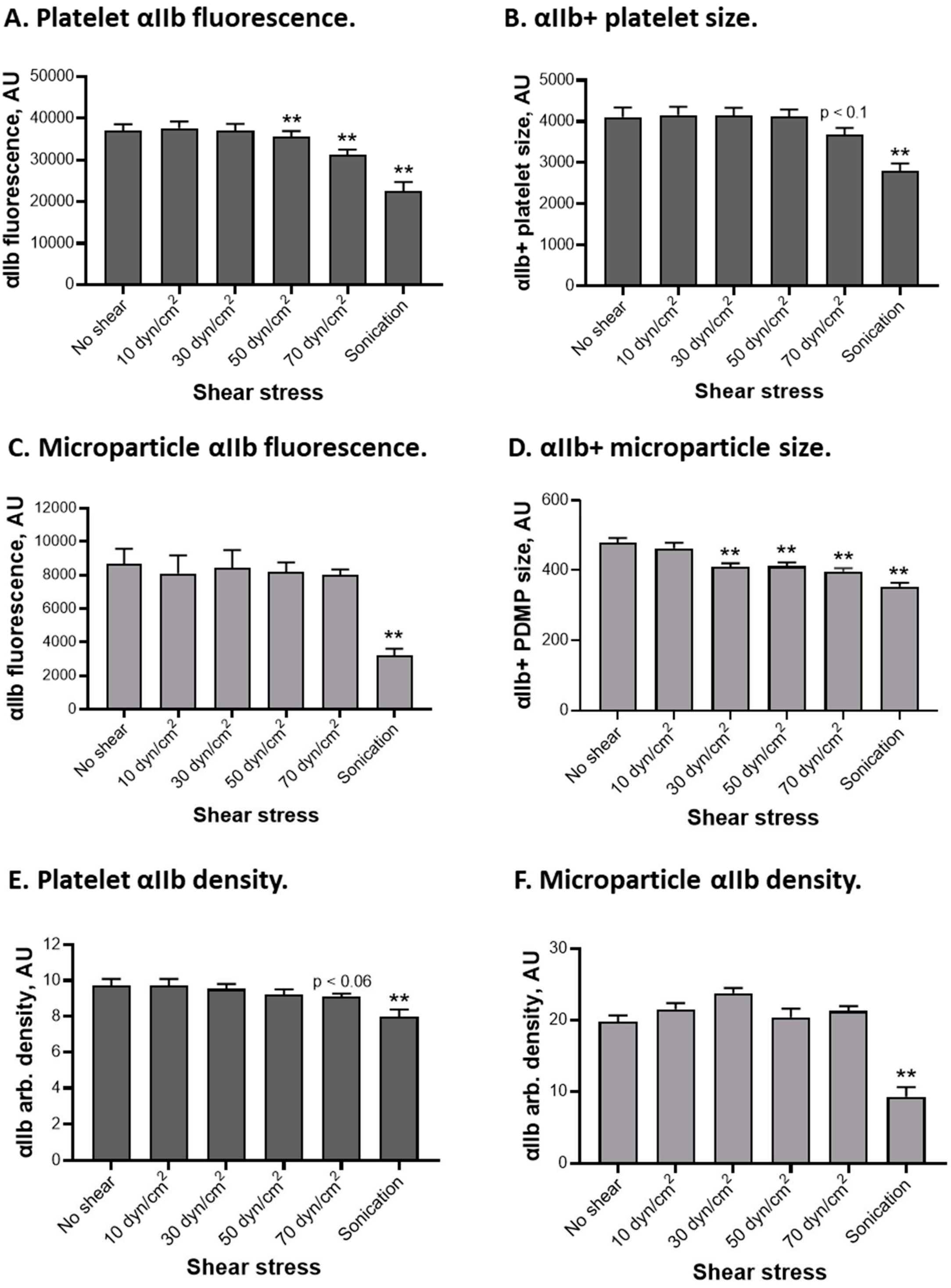

Figure S2. Distribution of αIIbβ3 integrin on platelets and platelet-derived microparticles (PDMPs): A, C – CD41 fluorescence on platelets and PDMPs, B, E - platelet and PDMP size (MFS), E, F – arbitrary density of αIIbβ3 integrin on platelets and PDMPs. N = 4-6. Mean ± SEM, 1-way ANOVA followed by Dunnett multiple comparisons test: ** - p<0.01 vs no shear.

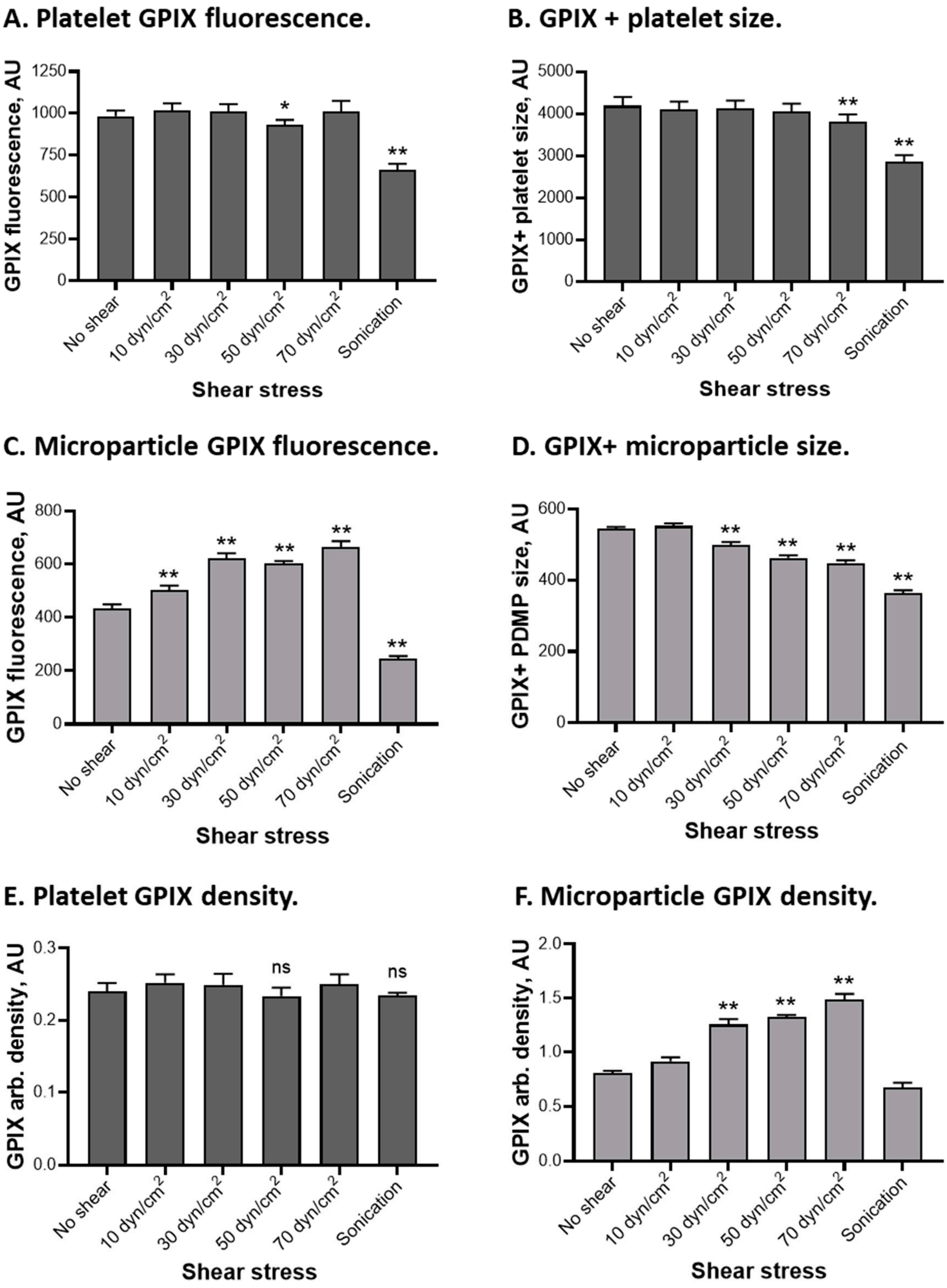

<u>Figure S3</u>. Distribution of GPIX on platelets and platelet-derived microparticles (PDMPs): A, C – CD42a fluorescence on platelets and PDMPs, B, E - platelet and PDMP size (MFS), E, F – arbitrary density of GPIX on platelets and PDMPs. N = 4-6. Mean ± SEM, 1-way ANOVA followed by Dunnett multiple comparisons test: * - p<0.05, ** - p<0.01, ns – nonsignificant vs no shear.

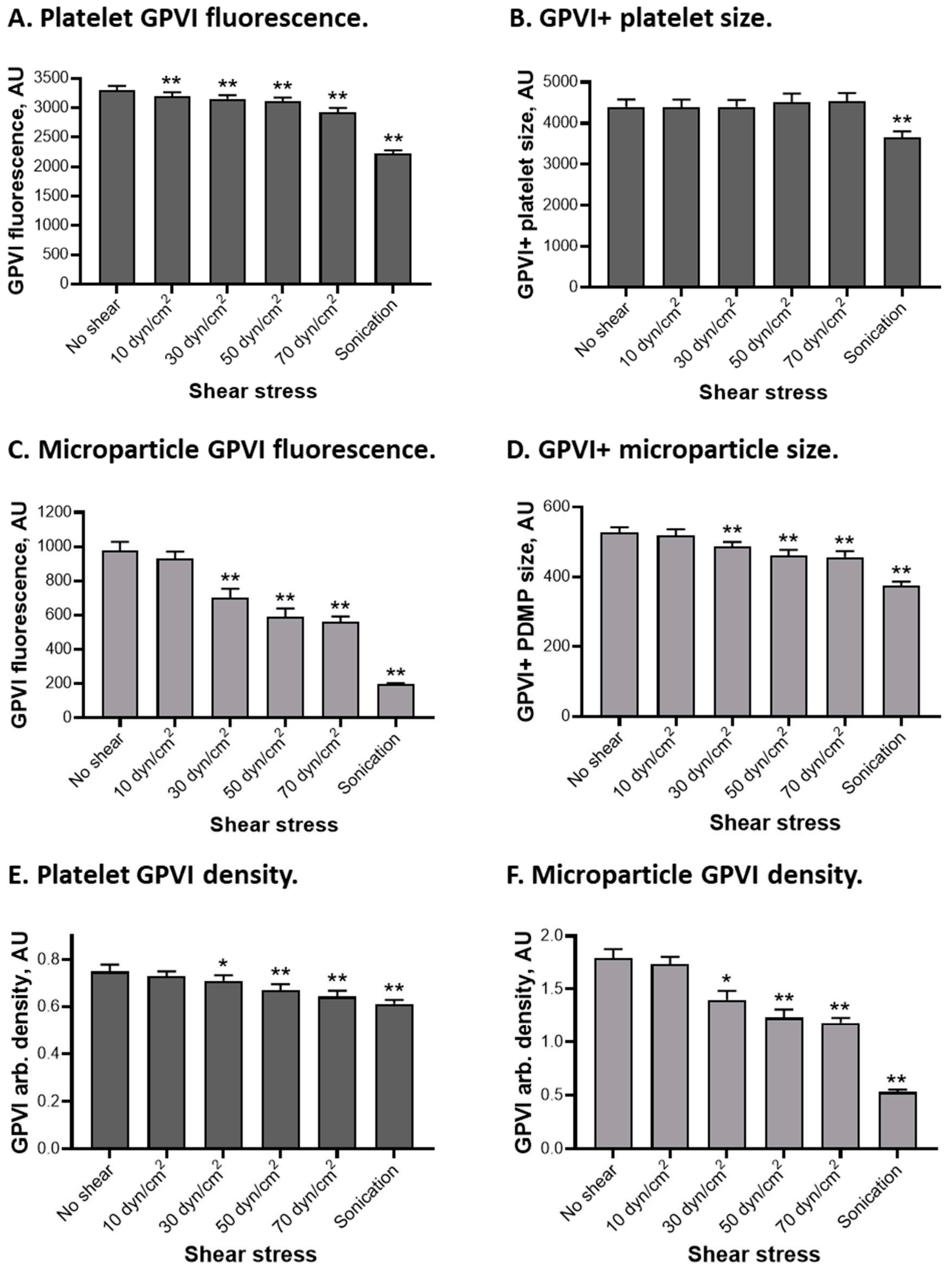

Figure S4. **Distribution of GPVI on platelets and platelet-derived microparticles (PDMPs):** A, C – GPVI fluorescence on platelets and PDMPs, B, E - platelet and PDMP size (MFS), E, F – arbitrary density of GPIX on platelets and PDMPs. N = 6-7. Mean ± SEM, 1-way ANOVA followed by Dunnett multiple comparisons test: * - *p*<0.05, ** - *p*<0.01 vs no shear.

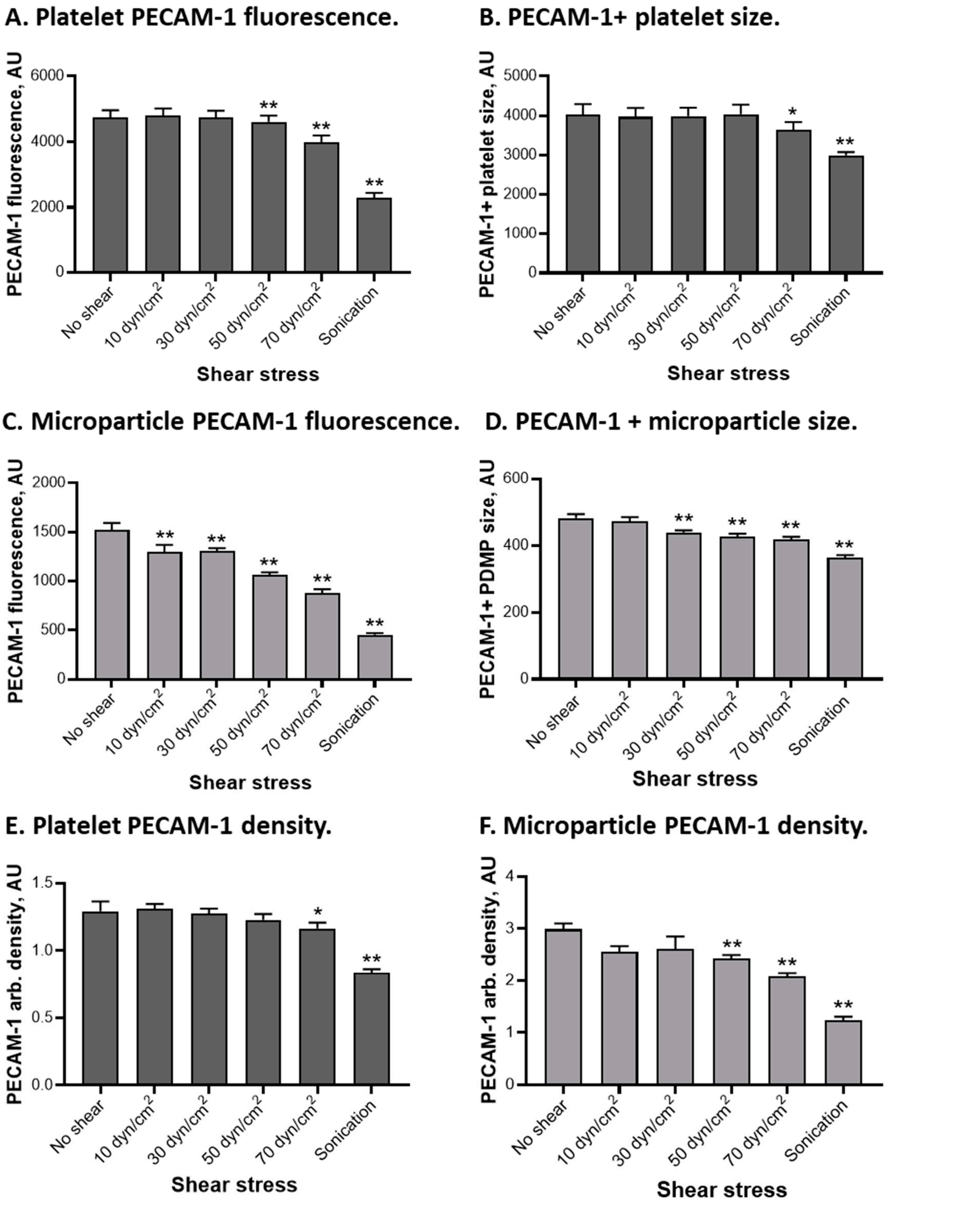

Figure S5. Distribution of PECAM-1 on platelets and platelet-derived microparticles (PDMPs): A, C – CD31 fluorescence on platelets and PDMPs, B, E - platelet and PDMP size (MFS), E, F – arbitrary density of GPIX on platelets and PDMPs. N = 6-7. Mean ± SEM, 1-way ANOVA followed by Dunnett multiple comparisons test: * - $p<0.05$, ** - $p<0.01$ vs no shear.

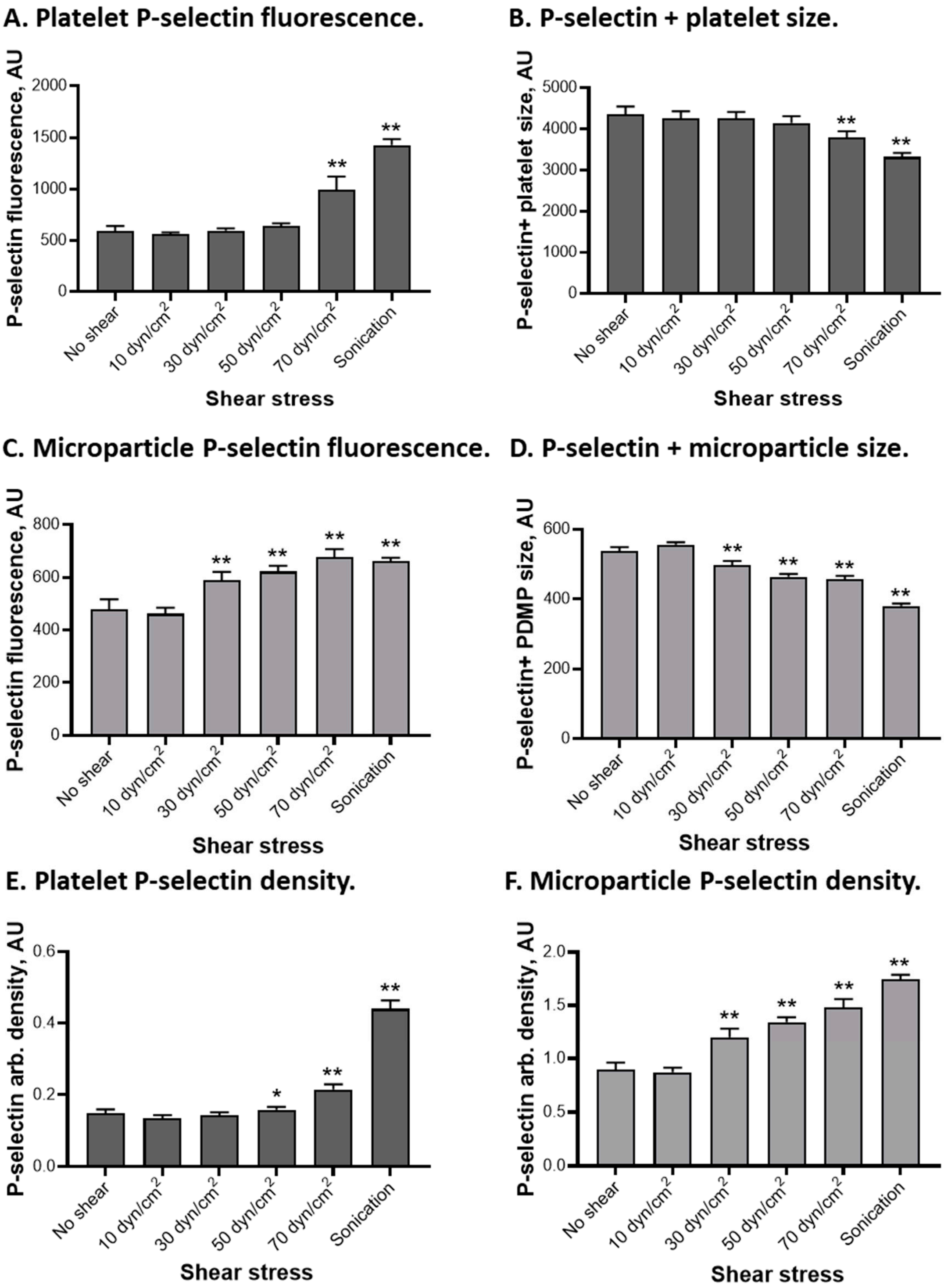

<u>Figure S6</u>. Distribution of P-selectin on platelets and platelet-derived microparticles (PDMPs): A, C – CD62P fluorescence on platelets and PDMPs, B, E - platelet and PDMP size (MFS), E, F – arbitrary density of GPIX on platelets and PDMPs. N = 6-7. Mean ± SEM, 1-way ANOVA followed by Dunnett multiple comparisons test: * - p<0.05, ** - p<0.01 vs no shear.

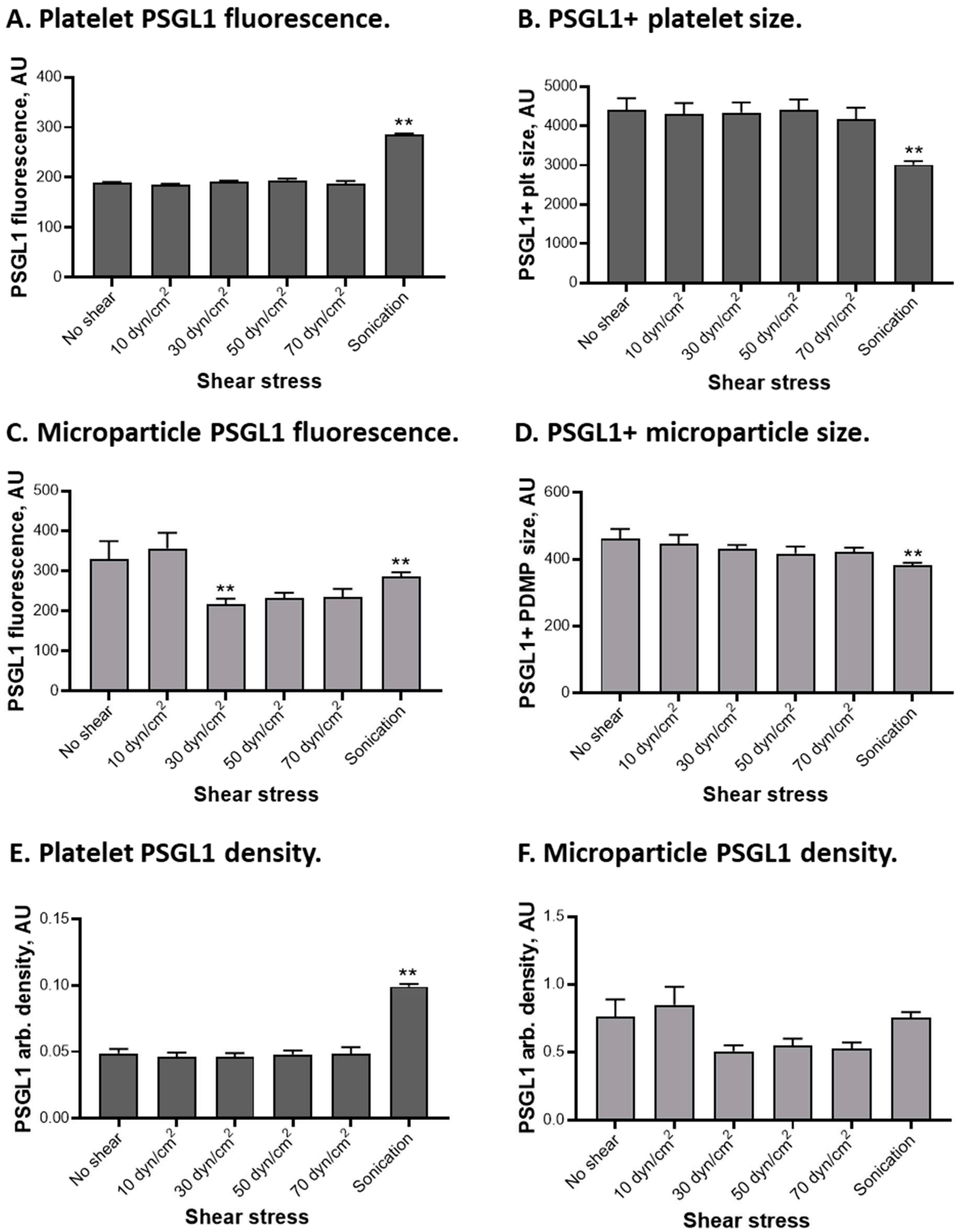

<u>Figure S7</u>**. Distribution of PSGL1 on platelets and platelet-derived microparticles (PDMPs):** A, C – PSGL1 fluorescence on platelets and PDMPs, B, E - platelet and PDMP size (MFS), E, F – arbitrary density of GPIX on platelets and PDMPs. N = 6-7. Mean ± SEM, 1-way ANOVA followed by Dunnett multiple comparisons test: ** - p<0.01 vs no shear.

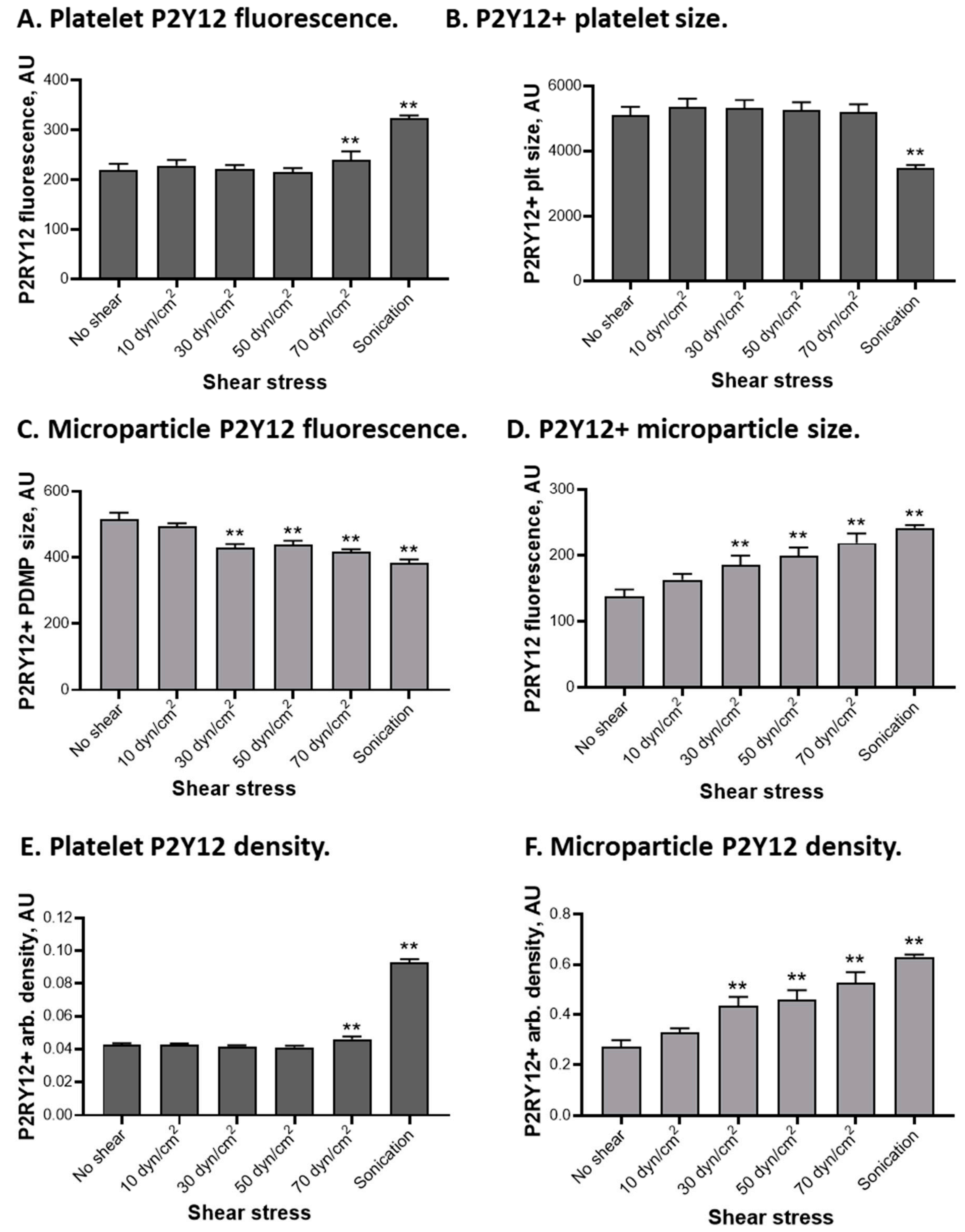

Figure S8. Distribution of P2Y12 on platelets and platelet-derived microparticles (PDMPs): A, C – P2RY12 fluorescence on platelets and PDMPs, B, E - platelet and PDMP size (MFS), E, F – arbitrary density of GPIX on platelets and PDMPs. N = 4-7. Mean ± SEM, 1-way ANOVA followed by Dunnett multiple comparisons test: ** - *p*<0.01 vs no shear.

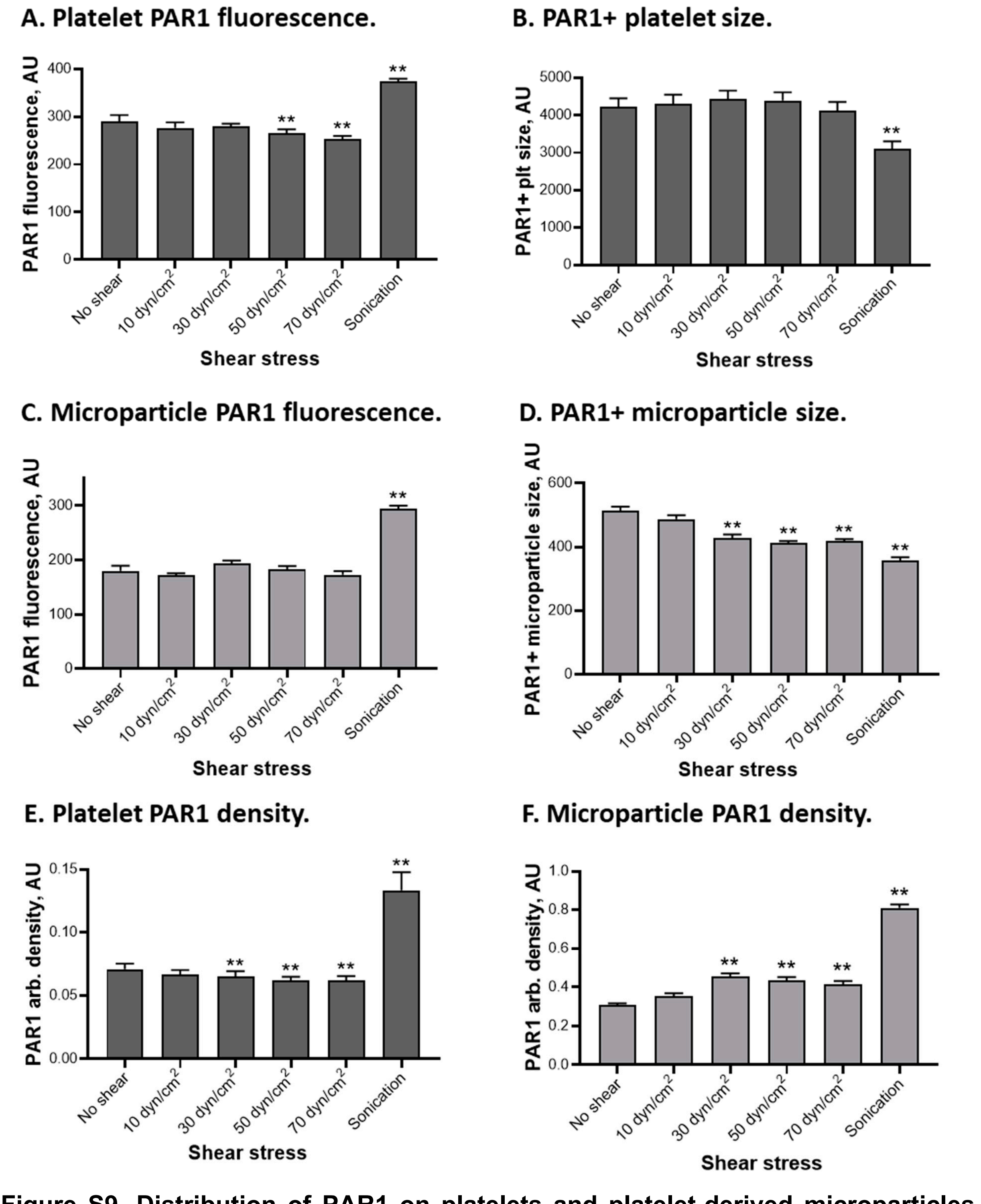

Figure S9. Distribution of PAR1 on platelets and platelet-derived microparticles (PDMPs): A, C – PAR1 fluorescence on platelets and PDMPs, B, E - platelet and PDMP size (MFS), E, F – arbitrary density of GPIX on platelets and PDMPs. N = 4-7. Mean ± SEM, 1-way ANOVA followed by Dunnett multiple comparisons test: ** - p<0.01 vs no shear.

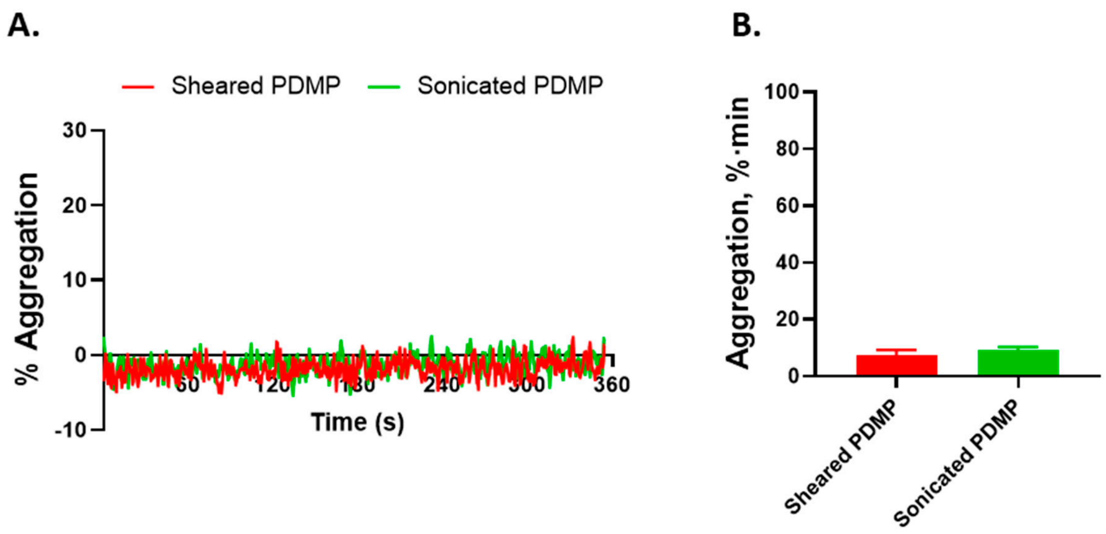

<u>Figure S10</u>. Platelet-derived microparticles (PDMPs) generated as a result of shear stress exposure and sonication do not induce platelet aggregation in plasma: A – representative aggregation curves; B – platelet aggregation, when no agonist was added (N = 18). Mean ± SEM, 1-way ANOVA followed by Dunnett multiple comparisons test.
